# Oxytocin infusion for maintenance of uterine tone under prophylactic phenylephrine infusion for prevention of post-spinal hypotension in cesarean delivery: a prospective randomised double-blinded dose-finding study

**DOI:** 10.1186/s12884-023-06165-5

**Published:** 2023-12-06

**Authors:** Xiao-Qin Jin, Yao-Hua Shen, Fan Fu, Juan Yu, Fei Xiao, Xiao-Dong Huang

**Affiliations:** 1 Department of Anesthesia, Hangzhou City Linping District Maternal and Child Care Hospital, Hangzhou, China; 2 Department of Anesthesia, Jiaxing University Affiliated Women and Children Hospital, Jiaxing, China; 3 Department of Anesthesia, Hangzhou Women’s Hospital (Hangzhou Maternity and Child Health Care Hospital, Hangzhou First People’s Hospital Qianjiang New City Campus, Zhejiang Chinese Medical University), Hangzhou, China

**Keywords:** Phenylephrine, Oxytocin, Cesarean delivery, Uterine tone, Hypotension

## Abstract

**Background:**

Prior studies have shown that, when administered as an intravenous bolus to prevent uterine atony, prophylactic phenylephrine infusion increased the dose requirement of oxytocin and second-line uterotonics. For the prevention of uterine atony, oxytocin should be delivered by continuous infusion. Here, we aimed to determine the ED50 and ED90 parameters (the effective doses for 50 and 90% patients without uterine atony) of oxytocin for co-infusion with prophylactic phenylephrine during cesarean delivery.

**Methods:**

In this prospective randomized double-blinded dose-finding study, one hundred patients were divided into four groups to receive 2.5, 5.0, 7.5, or 10 IU/h oxytocin infusion, after the umbilical cord was clamped during the study period. The uterine tone was evaluated and defined as either adequate or inadequate. Probit regression analysis was applied to calculate the ED50 and ED90 of oxytocin infusion. Uterine tone, the percentage of patients who needed additional oxytocin bolus, second-line uterotonics, side effects, estimated blood loss, and neonatal outcomes were monitored.

**Results:**

The estimated ED50 and ED90 values of the oxytocin infusion doses for the prevention of uterine atony were 1.9 IU/h (95% CI -4.6-3.8) IU/h and 9.3 IU/h (95% CI 7.3–16.2) IU/h, respectively. Across groups, there was a significant linear trend between the infusion dose and the percentage of patients who required additional oxytocin (*p*-value *=* 0.002). No differences were observed in the incidence of side effects and neonatal outcomes.

**Conclusion:**

Under the conditions of this study, the ED90 of oxytocin infusion for the prevention of uterine atony was 9.3 IU/h, which is higher than the current recommendation. This finding is helpful for clinical practice, because of the routine use of phenylephrine in cesarean delivery. Further studies are needed to determine the appropriate initial bolus of oxytocin after neonatal delivery.

**Trial registration:**

The study was registered on the Chinese Clinical Trial Register (register no. ChiCTR2200059556).

## Background

Postpartum haemorrhage (PPH) continues to be a significant contributor to maternal mortality. Oxytocin has been commonly used for the prevention of PPH. The optimal dosage and administration method of oxytocin utilized in clinical practice remains uncertain. In the past, 5 international unit (IU) oxytocin used to be a reasonable dose for preventing uterine atony [1, 2]. However, some clinical studies suggested lower dose of oxytocin is effective for maintaining uterine tone in CD [3–5]. In addition, oxytocin infusion during cesarean delivery is currently recommended to reduce the need for additional uterotonic agents, while maintaining the overall incidence of significant obstetric hemorrhage unchanged [6]. Therefore, an international consensus statement has recently recommended oxytocin administration as an initial bolus of 1 IU in 15 seconds, and followed by its infusion at a rate of 2.5–7.5 IU/h for elective cesarean delivery [7]. Many factors can influence the oxytocin dose required for maintaining uterine tone (UT) in cesarean delivery, including intrapartum delivery, obesity, and preeclampsia [8–10]. Thus, the recommendation is not suitable for all kinds of population.

In the prevention or treatment of post-spinal hypotension, prophylactic phenylephrine administration as an infusion has shown superior efficacy to its bolus administration. This is because its use is associated with lower incidence of hypotension, nausea, and vomiting [11–13]. Our previous study showed that a prophylactic phenylephrine infusion to prevent post-spinal hypotension increased the dose requirement of oxytocin and second-line uterotonics during cesarean delivery [14]. Nevertheless, oxytocin, because of its pharmacokinetic properties, is recommended to be delivered by continuous infusion following a bolus dose. For patients undergoing cesarean delivery under prophylactic phenylephrine infusion, the ideal oxytocin infusion dose is still unclear. In addition, the utilization of oxytocin is accompanied by numerous adverse effects, which restrict its application in certain female individuals, particularly those with severe cardiovascular conditions. To mitigate these unfavorable impacts, it is crucial to use the minimal efficacious dosage for attaining sufficient uterine contraction.

In the current study, we aimed to determine the 90% effective dose (ED90, the effective dose that prevents uterine atony in 90% of patients) of oxytocin for infusion during cesarean delivery in patients receiving prophylactic phenylephrine. Our hypothesis was that the oxytocin infusion rate would be higher in patients receiving prophylactic phenylephrine than the rate recommended by the international consensus statement [7].

## Methods

### Ethics

The present study was approved by the Ethical Committee of Hangzhou City Linping District Maternal and the Child Care Hospital, Hangzhou, China (approval no. LLSC-KYKT-2022-0007-A, Chairperson: Prof. Yue-Jian Shen) on March 23, 2022. The study was registered on the Chinese Clinical Trial Register (register no. ChiCTR2200059556) on 04/05/2022. All patients involved in this study provided written informed consent. The first patient was enrolled in this study on May 11, 2022, and the study was concluded on January 16, 2023.

### Design

This is a prospective randomized double-blinded dose-finding study.

### Patients and setting

One hundred patients with singleton, full-term pregnancies, scheduled for elective cesarean delivery, were enrolled in this study. Exclusion criteria were: patients with American Society of Anesthesiologists physical status ≥ III, obesity (body mass index, BMI > 35 kg/m^2^), diabetes mellitus or gestational diabetes, hypertension or pre-eclampsia, intrauterine growth restriction or fetal macrosomia, placenta previa, placenta conglutination, placenta implantation (if found during surgery), transverse lie, multiple gestation, fibroids, previous history of PPH, significant co-existing maternal disease, and any absolute contraindications to spinal or epidural anesthesia, such as local infection or bleeding disorders.

### Study protocol

Based on a randomized number sheet generated by a research assistant using an online randomization generator (https://www.random.org/sequences/), according to previous report [7],which suggested oxytocin infusion at a rate of 2.5–7.5 IU/h for elective cesarean delivery, patients were allocated into one of four groups to receive 2.5, 5.0, 7.5, or 10 IU/h oxytocin infusion. Then, the randomized number sheet was hidden in numbered opaque envelopes and opened after the corresponding parturient enrolled. Oxytocin was prepared under sterile conditions, in identical 50-ml infusion syringes based on the randomization sheet by a permanent anesthesiologist (Dr. Yao-Hua Shen), who was aware of patient grouping but was not involved in patient management and data collection. Patients involved in this study were not aware of the oxytocin dose they would receive.

None of the patients received any premedication. In the waiting room, peripheral access was created using an 18 G intravenous cannula inserted into the left upper limb vein. Upon arrival at the operating room, a standard monitoring procedure, which included a non-invasive blood pressure cuff, electrocardiography leads, and a pulse oximeter, was applied to record the patients’ vital signs. The baseline systolic blood pressure (SBP) and heart rate (HR) were calculated as the means of three readings, which were consecutively measured at 3-min intervals, after the patient was relaxed. Oxygen was delivered by a nasal catheter at a rate of 3 L per minute. There was no prehydration.

With the patient positioned on a left lateral position, the combined spinal-epidural technique was performed using the needle-through-needle procedure at the established L3-L4 disc space, which was detected by palpation. After detecting cerebrospinal fluid (CSF) efflux, 15 mg hyperbaric ropivacaine combined with 5 μg sufentanil were injected into the subarachnoid space over 20 seconds. Immediately after intrathecal injection, 0.5 μg/kg/min phenylephrine [15] were infused, followed by 10 ml/kg warmed (37 °C) Lactated Ringer’s solution intravenous administration over 20 minutes.

According to institutional standards, hypotension was defined as a fall of SBP ≥ 20% from its baseline value and to an absolute value of SBP < 90 mmHg. Hypertension was defined as an absolute value of SBP > 140 mmHg. Bradycardia was defined as the HR < 55 beats per minute (bpm). If hypotension was accompanied by a rapid HR (> 90 bpm), 100 μg phenylephrine were administered by an intravenous (i.v.) injection. If hypotension was not accompanied by a rapid HR, 5 mg ephedrine were administered through i.v. injection. If bradycardia co-occurred with hypotension, it was treated by an i.v. injection of 0.5 mg atropine. If not, phenylephrine infusion was paused and restarted when the HR > 55 bpm.

Surgery was not permitted until the sensory block level (by asking the patient for the loss of pinprick sensation) reached T6 dermatome or higher. All cesarean procedures were accomplished by the same group of surgeons with more than 10 years of obstetric experience. After neonatal delivery and umbilical cord clamping, 3 IU oxytocin were slowly intravenously injected over 15 seconds, followed by an infusion of 50 mL/h oxytocin (whose dose depended on the patient’s group) to prevent uterine atony. Until 20 minutes after umbilical cord clamping, the adequacy of UT was assessed by the leader of the obstetric group at 3-min intervals [16]. According to our study protocol, if the UT was found to be inadequate 3 min after the initial bolus, another 3 IU of oxytocin was administered by i.v. injection. If, after two oxytocin boluses, the UT was still found to be inadequate, second-line uterotonics (0.25 mg of intramuscular carboprost or 0.1 mg of intravenous carbetocin, which was based on obstetrician’s request) were administered as needed.

### Measurements

The primary outcome of the current study was the adequacy of UT during the study period. The UT was ranked as most satisfied (touched as forehead), satisfied (touched as tip of nose), dissatisfied (touched as lip), and assessed by the obstetrician, who did not aware of patients’ group, at 5-min intervals till to close the peritoneum. Most satisfied and satisfied UT were regarded as adequate UT, dissatisfied UT was regarded as inadequate UT. The secondary outcomes were the proportion of patients who needed additional oxytocin bolus and second-line uterotonics, side effects after administration of oxytocin (including hypertension, hypotension, tachycardia, bradycardia, nausea, flushing, chest pain, and shivering), the estimated blood loss (EBL, estimated by visual assessment of suction bottles and drapes), and neonatal outcomes. Demographic characteristics (such as age, height, weight, and gestational age), surgery time, cesarean history, and volume of intravenous crystalloid were also recorded.

### Sample size calculation

Sample size was calculated using the Cochran-Armitage Test for the trend in proportions using PASS®, (Version 11.0.7, NCSS, LLC, Kaysville, UT, USA), in accordance with the primary outcome of this study. According to a pilot study for the 4 groups with infusion doses of 2.5, 5.0, 7.5, and 10 IU/h, the proportions of adequacy UT were 45, 61, 74, and 90%, respectively. The full sample of 76 subjects achieved 90% power to detect a linear trend using a two-sided Z-test and a significance level of 0.05000. To account for any potential dropouts (estimated as 20% of the total patients), the sample size was increased to 25 patients in each group.

### Statistical analysis

Analyses were performed using IBM SPSS Statistics for Windows version 22.0 (IBM Corp, Armonk, NY, USA), GraphPad Prism version 5.0 (GraphPad Software Inc., San Diego, CA, USA), and Microsoft Excel (Microsoft Corporation, Redmond, WA, USA). We assessed the distribution of the continuous variables using graphical displays of the data and the Kolmogorov-Smirnov test. Demographic data characteristics were presented using descriptive statistics. To evaluate baseline imbalance in the randomization of the four infusion doses, the absolute standardized difference (ASD) between each pair among the four infusion doses were calculated for each parameter. The maximum ASD among all pairs was identified. Imbalance was estimated as the maximum ASD being greater than the value calculated by the following equation (based on the recommendation for small sample sizes by Austin [17]):

$$ASD>1.96\times \surd \left(\left(n1+n2\right)\div n1n2\right)$$

where n1 and n2 are the per-group sample sizes for the pair.

For normally distributed data (including demographic data), the volume of i.v. crystalloid and umbilical arterial pH values were presented as the mean and standard deviation (SD). One-way analysis of variance (ANOVA) was used to assess whether all the means were identical. Then, *post-hoc* Bonferroni tests were used for pairwise group comparison. Non-normally distributed data, including surgery times, EBL, Apgar score, and umbilical arterial pH and base excess (BE), were presented as the median and quartiles. The Kruskal-Wallis test was used to compare the groups on data distribution, with *post-hoc* Dunn’s tests for pairwise group comparisons. For categorical data, including rescue oxytocin administration and the incidence of side effects, the Cochran-Armitage χ2 test was used to assess linear trends in the parameters across the ordered randomized infusion dose groups. The dose-response relationship between the infusion dose and the UT was estimated by probit regression. The Pearson goodness-of-fit chi-square test was used to test the null hypothesis that the probit model adequately fitted the data. Where Bonferroni corrections were applied, adjusted *p*-values were presented. Two-tailed *p-*values < 0.05 were regarded as statistically significant.

## Results

**Fig. 1:**
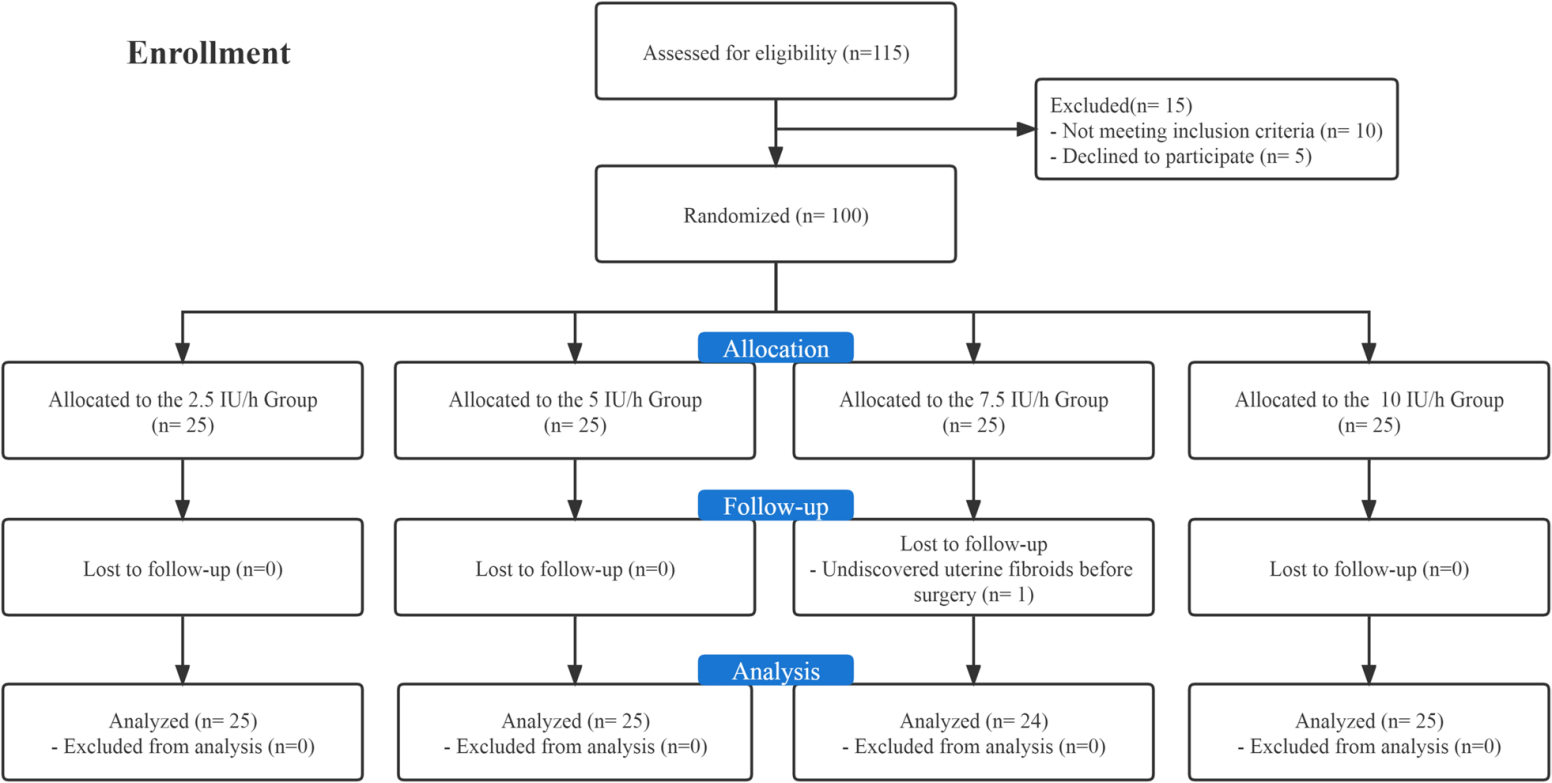
CONSORT flow diagram

A hundred and twelve participants were recruited and assessed for eligibility to participate in the present study. Of those, eight patients declined to participate, and four patients did not meet the inclusion criteria. A hundred patients were allocated into four infusion dose groups. One patient in group 7.5 was excluded because of a previously undiscovered uterine fibroid condition. Data from ninety-nine participants were utilized for the final analysis (Fig. 1). Patient demographic data are shown in Table 1.

**Table 1:** Demographics, surgery and preoperative Hb and HCT data

	Oxytocin infusion rates				
Characteristic	2.5 IU/h (*n* = 25)	5.0 IU/h (*n* = 25)	7.5 IU/h (*n* = 24)	10.0 IU/h (*n* = 25)	Maximum ASD between groups
Age (year)	30.5 ± 3.9	30.1 ± 4.1	30.5 ± 3.9	30.0 ± 4.3	0.12 (0.55)
Height (cm)	159 ± 5	161 ± 4	161 ± 3	161 ± 5	0.49 (0.56)
Weight (kg)	66 ± 8	69 ± 8	67 ± 7	70 ± 7	0.53 (0.55)
Gestational age (week)	38.4 ± 1.0	38.5 ± 0.8	38.5 ± 1.1	38.6 ± 0.9	0.21 (0.55)
Surgery time (min)	47.6 ± 18.3	46.0 ± 16.5	49.6 ± 12.2	45.7 ± 14.1	0.30 (0.56)
Cesarean history (%)	11 (44)	12 (48)	15 (63)	12 (48)	0.13 (0.55)
Preoperative Hb (g/dL)	126.4 ± 9.9	124.2 ± 11.7	125.2 ± 9.2	122.5 ± 12.9	0.34 (0.55)
Preoperative HCT (%)	39.2 ± 3.7	38.4 ± 3.3	38.3 ± 2.8	38.2 ± 4.5	0.24 (0.55)

Data shown as number (%), or mean (SD) as appropriate

**Fig. 2:**
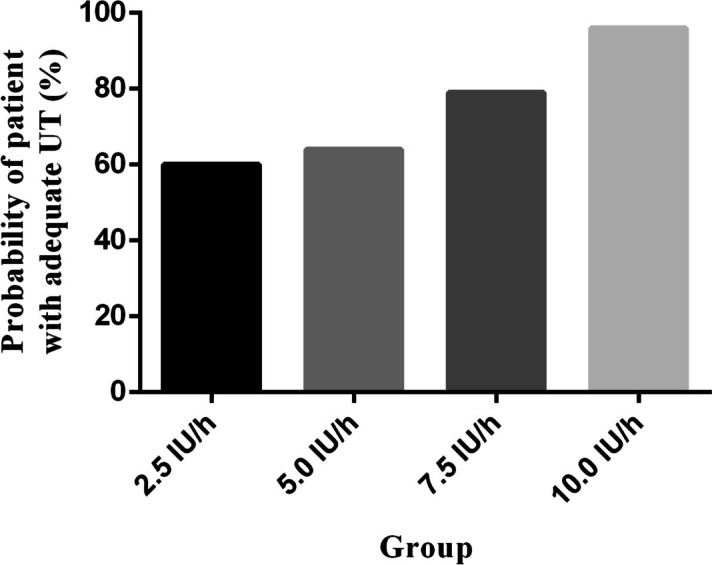
Proportion of patients with adequate uterine tone at corresponding infusion dose of oxytocin

**Fig. 3:**
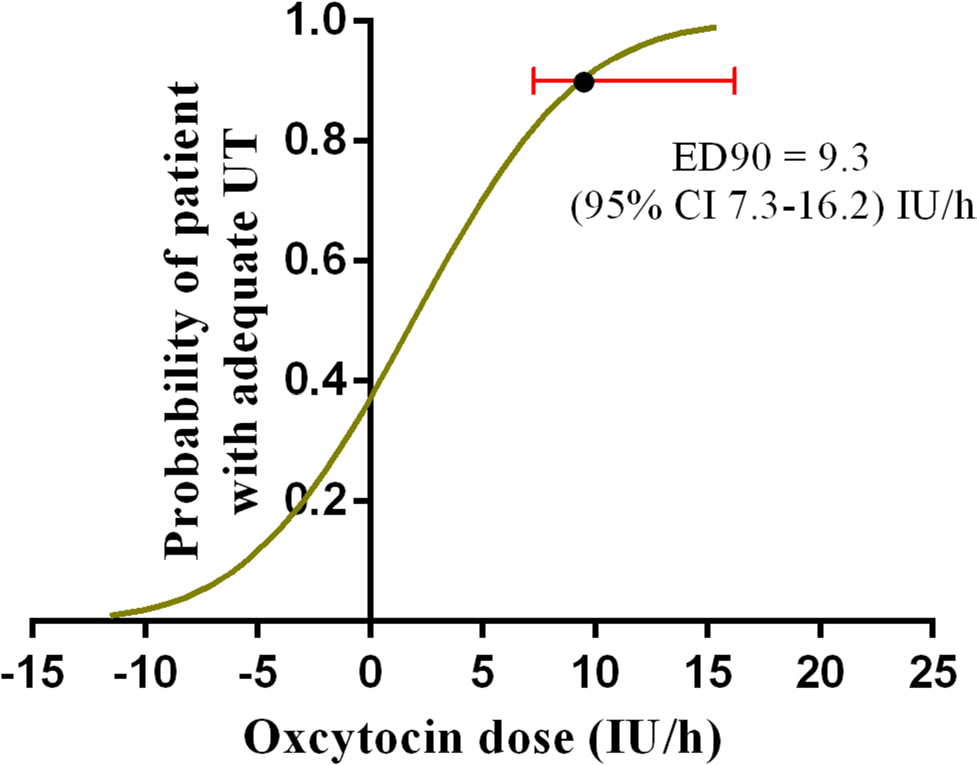
Dose-response curve of oxytocin at corresponding infusion dose (IU/h) for prevention uterine atony. The estimated values of ED50 and ED90 of the infusion dose of oxytocin for the prevention of uterine atony were 1.9 IU/h (95% CI: − 4.6 -3.8) IU/h and 9.3 IU/h (95% CI 7.3–16.2) IU/h, respectively

The percentages of adequate UT in the setting of the four infusion doses of oxytocin, followed by a bolus of 3 IU oxytocin, were 60, 64, 79, and 96%, respectively (Fig. 2). The estimated values of ED50 and ED90 of the infusion dose of oxytocin for the prevention of uterine atony were 1.9 IU/h (95% CI: -4.6-3.8) IU/h and 9.3 IU/h (95% CI 7.3–16.2) IU/h, respectively, which were derived from probit analysis. The results of the probit regression are shown in Fig. 3. The results of the Pearson goodness-of-fit chi-square test showed that the probit model fitted well (*p* = 0.428).

Secondary outcome data are presented in Table 2. A total of 40% (10/25), 36% (9/25), 21% (5/24), and 4% (1/25) patients in groups 2.5, 5.0, 7.5, and 10, respectively, received rescued oxytocin. There was a significant linear trend between the infusion dose and the percentage of patients who required rescued oxytocin across groups (*p =* 0.002). There were four patients in group 2.5, three in group 5.0, two in group 7.5, and two in group 10.0 who needed second-line uterotonics after two boluses of oxytocin. There were no significant differences among groups (*p* = 0.143). There were also no significant differences among groups in the values of the EBL (*p* = 0.456). There were also no significant differences in the Hb and HCT values, which were checked during the preoperative day and postoperative day 1. Among the groups, there were no patients who experienced PPH.

**Table 2:** Secondary outcomes

	Oxytocin infusion rates				
	2.5 IU/h (*n* = 25)	5.0 IU/h (*n* = 25)	7.5 IU/h (*n* = 24)	10.0 IU/h (*n* = 25)	*P* value
Rescue oxytocin required	10 (40)^a^	9 (36)^b^	5 (21)	1 (4)	0.002
Secondary uterine agent	4 (16)	3 (12)	2 (8)	1 (4)	0.143
Hb on postoperative day 1 (g/dL)	119.1 ± 10.5	114.8 ± 10.0	118.0 ± 9.6	115.9 ± 17.3	0.586
HCT on postoperative day 1 (%)	35.5 ± 2.7	35.5 ± 3.0	35.2 ± 3.0	34.8 ± 4.8	0.889
EBL during surgery (ml)	570 (450–795)	590 (495–870)	540(388–698)	560(435–820)	0.456
EBL calculated from HCT (ml)	517 ± 429	430 ± 401	447 ± 349	527 ± 428	0.779

Data shown as number (%), median (quartiles), or mean (SD) as appropriate

^a^*p =* 0.0046 vs 10, ^b^*p =* 0.0106 vs versus group 10

Side effects after administration of oxytocin and neonatal outcomes are presented in Table 3. Concerning the incidence of side effects and neonatal outcomes, no significant differences were observed among the groups.

**Table 3:** Side effects and neonatal outcomes

	Oxytocin infusion rates				
	2.5 IU/h (*n* = 25)	5.0 IU/h (*n* = 25)	7.5 IU/h (*n* = 24)	10.0 IU/h (*n* = 25)	*P* value
Hypertension	0 (0)	0 (0)	1(4)	2 (8)	0.068
Hypotension	5 (20)	5 (20)	5 (21)	3 (12)	0.502
Bradycardia	0 (0)	1 (4)	2 (8)	1 (4)	0.361
Nausea	2 (8)	0 (0)	1 (4)	1 (4)	0.658
Flushing	2(8)	1(4)	2 (8)	3 (12)	0.505
Shivering	0 (0)	0 (0)	0 (0)	1 (4)	0.180
Chest pain	1 (4)	0 (0)	1 (4)	1(4)	0.995
1-min Apgar score	9 (9–10)	9 (9–10)	10 (9–10)	10 (9–10)	0.527
5-min Apgar score	10 (10–10)	10 (10–10)	10 (10–10)	10 (10–10)	0.899
Umbilical arterial pH	7.31(7.28–7.35)	7.31(7.29–7.35)	7.31(7.28–7.33)	7.31(7.29–7.34)	0.989
Umbilical arterial PO_2_	20.8 ± 4.7	20.4 ± 6.3	22.7 ± 7.0	23.0 ± 7.8	0.384
Umbilical arterial PCO_2_	45.6 ± 8.6	47.8 ± 6.8	50.4 ± 10.5	51.6 ± 11.1	0.107
Umbilical arterial BE	−2.1(−6.0 - -1.0)	−2.2(−5.6 - -0.6)	−0.9(−4.1–0.4)	−1.2(− 2.5–0.7)	0.131
Umbilical arterial HCO_3_^−^	23.3 (18.5–25.0)	24.3 (19.5–26.0)	26.0 (22.5–27.4)	25.6 (22.9–27.1)	0.061
Neonatal weight, g	3276 ± 279	3330 ± 380	3317 ± 321	3424 ± 267	0.403

Data shown as number (%), median (quartiles), or mean (SD) as appropriate

## Discussion

In this prospective dose-finding study, we have shown that, in women receiving 0.5 μg/kg/min phenylephrine to prevent post-spinal hypotension during elective cesarean delivery, the ED90 of oxytocin to prevent uterine atony was 9.3 IU/h. This value is significantly higher than the recommended dose by an international consensus statement [7]. The trend of increasing oxytocin infusion dose demand in patients with prophylaxis phenylephrine is consistent with our prior study using oxytocin bolus. In which, women receiving prophylactic phenylephrine required larger doses of oxytocin bolus than women without prophylactic phenylephrine treatment [14].

Oxytocin is widely used for the prevention of PPH. Various organizations have suggested different protocols for the use of oxytocin during the third stage of labor in cesarean delivery. The Royal College of Obstetricians and Gynaecologists of the UK has recommended 5 IU of oxytocin slowly infused as an i.v. bolus [18]. In contrast, the American College of Obstetricians and Gynecologists of the USA recommends 10 IU of oxytocin administered either as a dilute infusion or as an intramuscular injection [19]. For elective cesarean delivery, in 2019, an international consensus statement recommended the use of oxytocin as an initial 1 IU bolus in 15 seconds upon neonatal delivery, followed by an infusion (2.5–7.5 IU/h). Then, after UT assessment, an additional bolus of oxytocin or second-line uterotonics should be delivered [1].

However, many factors could influence the UT. In Lavoie’s study, it was found that women with prior exposure to exogenous oxytocin required higher infusion doses of oxytocin to maintain UT, in comparison with women without prior exposure (44.2 IU/h vs. 16.2 IU/h) [8]. Peska et al. conducted a clinical trial to compare the dose requirement of an initial bolus of oxytocin in patients with or without obesity. They found that women with a BMI ≥ 40 kg.m^−2^ required more than twice the oxytocin dose than women with a BMI < 40 kg.m^−2^ (0.78 vs. 0.35 IU) [9]. Tyagi et al. [10] compared the ED90 of prophylactic oxytocin infusion in patients with pre-eclampsia (who received magnesium therapy) and normotensive pregnant women. They found that the ED90 was significantly higher in patients who received magnesium sulfate than in patients who did not (24.9 IU/h vs. 13.9 IU/h). Prior studies demonstrated that a prophylactic phenylephrine infusion during cesarean delivery was another potential factor that could influence uterine contraction [14]. Nevertheless, under this condition, the ideal oxytocin infusion dose for preventing uterine atony was still unknown.

The strength of the present study is in providing full dose-response information on the oxytocin infusion dose for the prevention of uterine atony. In this study, the calculated ED90 (9.3 IU/h) of the oxytocin infusion dose was higher than the results of Qian’s study. In Qian’s study, it was found, using a very similar probit regression design to ours, that after a bolus of 1 IU oxytocin the ED95 infusion dose of oxytocin was 7.72 IU/h [20]. Duffield et al. [16] conducted a comparison between infusions of 2.5 IU/h and 15 IU/h following an initial bolus of 1 IU oxytocin. They found 2.5 IU/h as effective as 15 IU/h, which suggested that requiring infusion following an initial bolus necessitates the use of minimal dosages. Kovacheva et al. [21] found a bolus of 3 IU oxytocin following an infusion of 3 IU/h is noninferior to an “wide-open” infusion 500 mL contained 30 IU oxytocin for preventing uterine atony in cesarean delivery. Although a direct comparison is lacking, our results concerning the oxytocin infusion dose are numerically higher than those of previous studies. They are also higher than the dose recommended by the international consensus statement [7], which indicates that the oxytocin infusion dose needs to be increased when phenylephrine is infused, to prevent post-spinal hypotension in cesarean delivery. However, using a biased-coin design up-down sequential method, George et al. [22] found that the ED90 (95%CI) of an oxytocin infusion was 17.4 (9.0–25.8) IU/h for elective cesarean delivery. Similarly, using a same method, Lavoie et al. [8] found the ED90 of an oxytocin infusion was 16.2 (13.1–19.3) IU/h for non-laboring parturients. Different with their study, a loading dose of 3 IU oxytocin was intravenously administered before infusion, which would result in a low dose infusion in this study.

The exact mechanism through which prophylactic phenylephrine increases the oxytocin dose requirement to prevent uterine atony is still unclear. Nevertheless, there are two potential explanations. First, phenylephrine suppressed uterine contraction in non-pregnant mice, through cyclic adenosine monophosphate (cAMP) signaling via β2-receptor activation [23]. Similarly, increased cAMP levels were induced by phenylephrine in human uterine smooth muscle cells (HUSMCs), suggesting that phenylephrine would also exhibit antagonistic effects against contractions in the human uterus [23]. Second, phenylephrine infusion during surgery elevated blood pressure and subsequently increased uterine perfusion pressure, which could increase bleeding during the uterine incision procedure [14]. Therefore, clinical interventions, such as a rescuing dose of oxytocin, could be a possibility for mitigating the cause of bleeding.

It should be kept in mind that the optimum dose of the initial bolus of oxytocin is still unknown in patients with prophylactic phenylephrine treatment after neonatal delivery. We chose an initial dose regimen of 3 IU rather than 1 IU, (3-fold the dose suggested by the international consensus statement), to reduce the potential higher risk of PPH in patients receiving prophylactic phenylephrine to manage post-spinal blood pressure. In this framework, further studies are needed to determine the optimal initial dose of oxytocin. Notably, we would like to interpret that the estimated blood loss was similar among the four fixed infusion doses of oxytocin, due to the close observation and timely treatment with rescued oxytocin and the second line uterine agent. Hence, the data in this study suggested that the management of oxytocin infusion should be based on patient’s response, because not all patients respond to oxytocin in the same way.

It should be noted that oxytocin use is associated with major cardiovascular side effects, including hypotension, ST depression, and tachycardia. Despite seemingly less serious, nausea, vomiting, chest pain, headache, and flushing are additional commonly experienced and unpleasant side effects. After careful literature revision, we found that these side effects are dependent on the dose and the administration rate. A randomized clinical trial has shown that ST depression occurred in 8% of women after a 5 IU oxytocin bolus vs. 22% after 10 IU, which was proved to be related to the occurrence of severe hypotension [24]. Thomas et al. [25] observed that a slow administration of 5 IU oxytocin for 5 minutes led to decreased effects on the decrement in mean arterial pressure and in the increase in the heart rate, than the administration of the same dose as a bolus. We found no differences in the incidence of side effects among the groups in our study. Besides the factors mentioned above, the small sample size of the current study does not have sufficient power to determine the differences in the secondary outcomes among groups, which may be a reasonable explanation for this finding.

There were several limitations in the current study. First, the assessment of UT was subjective. Different obstetricians could have different evaluation criteria for the UT. Second, because of the critical criteria used for patient inclusion and exclusion, the results of the current may not apply to all kinds of patients. In addition, this study only enrolled patients for elective cesarean delivery; therefore, the ED90 value determined might not be applicable to patients under emergency cesarean delivery. Finally, the initial oxytocin dose in this study was 3 IU. Different institutions may use different initial bolus administration doses, which could influence the dose requirement for the subsequent infusion. Thus, further studies are needed to evaluate whether the ED90s of oxytocin infusion are changed when different initial boluses of oxytocin are used.

## Conclusions

In summary, under the conditions of this study, the ED90 of oxytocin infusion for the prevention of uterine atony was 9.3 IU/h following an initial bolus of 3 IU, which is higher than the current recommendations. This finding is helpful for clinical practice, because phenylephrine is routinely used in cesarean delivery. Further studies are needed to determine the appropriateness of the initial bolus of oxytocin after neonatal delivery.

## Data Availability

All data generated or analysed during this study are included in this published article.

## Abbreviations

ED90: 90% effective dose
PPH: postpartum hemorrhage
CD: cesarean delivery
UT: uterine tone
IU: international unit
BMI: body mass index
CSF: cerebrospinal fluid
SBP: systolic blood pressure
HR: heart rate
EBL: estimated blood loss
ASD: absolute standardized difference
SD: standard deviation
ANOVA: One-way analysis of variance

## References

[1] Wedisinghe L, Macleod M, Murphy DJ (2008). Use of oxytocin to prevent haemorrhage at caesarean section—a survey of practice in the United Kingdom. Eur J Obstet Gynecol Reprod Biol.

[2] King KJ, Douglas MJ, Unger W, Wong A, King RA (2010). Five unit bolus oxytocin at cesarean delivery in women at risk of atony: a randomized, double-blind, controlled trial. Anesth Analg.

[3] Carvalho JC, Balki M, Kingdom J, Windrim R (2004). Oxytocin requirements at elective cesarean delivery: a dose-finding study. Obstet Gynecol.

[4] Butwick AJ, Coleman L, Cohen SE, Riley ET, Carvalho B (2010). Minimum effective bolus dose of oxytocin during elective caesarean delivery. Br J Anaesth.

[5] Sartain J, Barry J, Howat P, McCormack D, Bryant M (2008). Intravenous oxytocin bolus of 2 units is superior to 5 units during elective caesarean section. Br J Anaesth.

[6] Sheehan SR, Montgomery AA, Carey M (2011). Oxytocin bolus versus oxytocin bolus and infusion for control of blood loss at elective caesarean section: double blind, placebo controlled, randomised trial. BMJ.

[7] Heesen M, Carvalho B, Carvalho JCA (2019). International consensus statement on the use of uterotonic agents during caesarean section. Anaesthesia.

[8] Lavoie A, McCarthy RJ, Wong CA (2015). The ED90 of prophylactic oxytocin infusion after delivery of the placenta during cesarean delivery in laboring compared with nonlaboring women: an up-down sequential allocation dose-response study. Anesth Analg.

[9] Peska E, Balki M, Maxwell C, Ye XY, Downey K, Carvalho JCA (2021). Oxytocin at elective caesarean delivery: a dose-finding study in women with obesity. Anaesthesia.

[10] Tyagi A, Mohan A, Singh Y, Luthra A, Garg D, Malhotra RK (2022). Effective dose of prophylactic oxytocin infusion during cesarean delivery in 90% population of nonlaboring patients with preeclampsia receiving magnesium sulfate therapy and normotensives: an up-down sequential allocation dose-response study. Anesth Analg.

[11] Ngan Kee WD (2017). The use of vasopressors during spinal anaesthesia for caesarean section. Curr Opin Anaesthesiol.

[12] Kinsella SM, Carvalho B, Dyer RA (2018). International consensus statement on the management of hypotension with vasopressors during caesarean section under spinal anaesthesia. Anaesthesia.

[13] George RB, McKeen DM, Dominguez JE, Allen TK, Doyle PA, Habib AS (2018). A randomized trial of phenylephrine infusion versus bolus dosing for nausea and vomiting during cesarean delivery in obese women. Can J Anaesth.

[14] Shen YH, Yang F, Jin LD (2021). Prophylactic phenylephrine increases the dose requirement of oxytocin to treat uterine atony during cesarean delivery: a double-blinded, single-center, randomized and placebo-controlled trial. Front Pharmacol.

[15] Xiao F, Shen B, Xu WP, Feng Y, Ngan Kee WD, Chen XZ (2020). Dose-response study of 4 weight-based phenylephrine infusion regimens for preventing hypotension during cesarean delivery under combined spinal-epidural anesthesia. Anesth Analg.

[16] Duffield A, McKenzie C, Carvalho B (2017). Effect of a high-rate versus a low-rate oxytocin infusion for maintaining uterine contractility during elective cesarean delivery: a prospective randomized clinical trial. Anesth Analg.

[17] Austin PC (2009). Balance diagnostics for comparing the distribution of baseline covariates between treatment groups in propensity-score matched samples. Stat Med.

[18] Prevention and Management of Postpartum Haemorrhage: green-top guideline no. 52. BJOG. 2017;124:e106–49. 10.1111/1471-0528.14178 27981719

[19] Postpartum hemorrhage (2017). Practice bulletin no. 183. American College of Obstetricians and Gynecologists. Obstet Gynecol.

[20] Qian XW, Drzymalski DM, Lv CC, Guo FH, Wang LY, Chen XZ (2019). The ED50 and ED95 of oxytocin infusion rate for maintaining uterine tone during elective caesarean delivery: a dose-finding study. BMC Pregnancy Childbirth.

[21] Kovacheva VP, Soens MA, Tsen LC (2015). A randomized, double-blinded trial of a “rule of threes” algorithm versus continuous infusion of oxytocin during elective cesarean delivery. Anesthesiology.

[22] George RB, McKeen D, Chaplin AC, McLeod L (2010). Up-down determination of the ED(90) of oxytocin infusions for the prevention of postpartum uterine atony in parturients undergoing cesarean delivery. Can J Anesth.

[23] Chen X, Meroueh M, Mazur G (2018). Phenylephrine, a common cold remedy active ingredient, suppresses uterine contractions through cAMP signalling. Sci Rep.

[24] Jonsson M, Hanson U, Lidell C, Nordén-Lindeberg S (2010). ST depression at caesarean section and the relation to oxytocin dose. A randomised controlled trial. BJOG.

[25] Thomas JS, Koh SH, Cooper GM (2007). Haemodynamic effects of oxytocin given as i.v. bolus or infusion on women undergoing caesarean section. Br J Anaesth.

